# Supplementary material for: Identification of photoperiod-induced specific miRNAs in the adrenal glands of Sunite sheep (Ovis aries)
Source: Front Vet Sci. 2022 Jul 22;9:888207. doi: 10.3389/fvets.2022.888207 (PMC9354845; doi:10.3389/fvets.2022.888207)
Supplement: Supplementary file 1 [file Data_Sheet_1.ZIP › Supplementary file/miRDeep2 file.zip!/miRDeep2 file/Novel_Structure/LP42A3/Novel_240.pdf]

Provisional ID	:	NC_019484.2_24943
Score total	:	1.8
Score for star read(s)	:	-1.3
Score for read counts	:	0
Score for mfe	:	1.5
Score for randfold	:	1.6
Score for cons. seed	:	
Total read count	:	87
Mature read count	:	87
Loop read count	:	0
Star read count	:	0

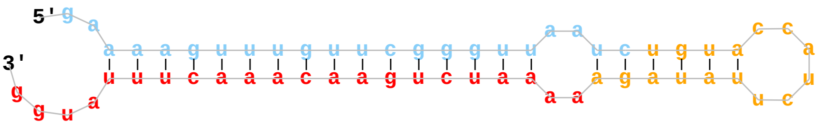

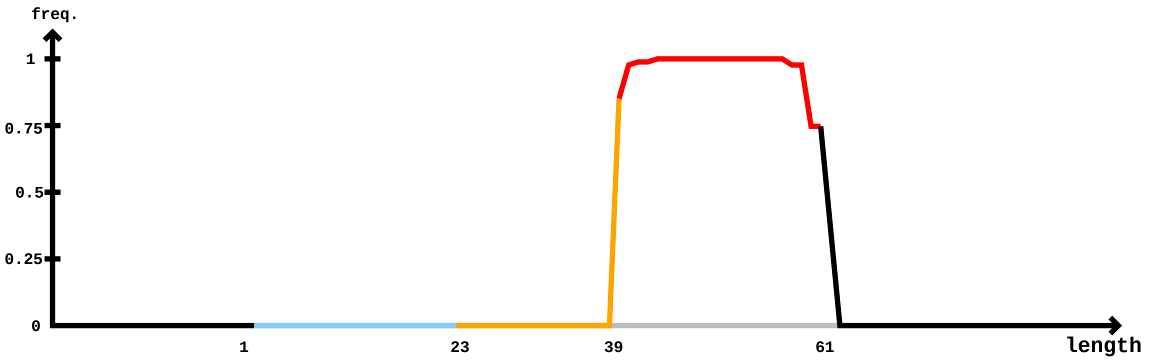


```
                     Star                                     Mature
5'- acaauccuagguguuggcugaaaaguuuguucggguuaaaucuguaccaucuuauagaaaaaucugaacaaacuuuaugg cuaacccaguauuuucucuucuuaccugaa   -3'    exp
    .........((.((((((((.(((((((((((((((((..((((((......))))))..)))))))))))))))).))))))))))).......................    reads    mm    sample
    ..........................................................aaaaCcugaacaaacuuu..................................    2        1     seq
    ..........................................................aaaaucugaacaaacuuuUu................................    20       1     seq
    ..........................................................aaaaucugaacaaacuuuUugg..............................    51       1     seq
    ..........................................................aaaaucugaacaaacuuuUuggc.............................    1        1     seq
    ...........................................................aaaucugaacaaacuuuUugg..............................    11       1     seq
    ............................................................aaucugaacaaacuuuUuggc.............................    1        1     seq
    ..............................................................ucugaacaaacuuuUuggc.............................    1        1     seq
```
